# The multikinase inhibitor regorafenib decreases angiogenesis and improves portal hypertension

**DOI:** 10.18632/oncotarget.26333

**Published:** 2018-11-16

**Authors:** Frank Erhard Uschner, Florian Schueller, Ivelina Nikolova, Sabine Klein, Robert Schierwagen, Fernando Magdaleno, Stefanie Gröschl, Sven Loosen, Thomas Ritz, Christoph Roderburg, Michael Vucur, Glen Kristiansen, Twan Lammers, Tom Luedde, Jonel Trebicka

**Affiliations:** ^1^ Department of Internal Medicine I, University of Bonn, Bonn, Germany; ^2^ Department of Internal Medicine III, University of Aachen, Aachen, Germany; ^3^ Institute of Cellular Medicine, Fibrosis Research Group, Newcastle upon Tyne, United Kingdom; ^4^ Department of Pathology, University of Bonn, Bonn, Germany; ^5^ Department of Nanomedicine and Theranostics, Institute for Experimental Molecular Imaging, University of Aachen, Aachen, Germany; ^6^ Institute of Clinical Research, Odense University Hospital, University of Southern Denmark, Odense, Denmark; ^7^ European Foundation for the Study of Chronic Liver Failure, Barcelona, Spain; ^8^ Institute for Bioengineering of Catalonia, Barcelona, Spain; ^9^ Department of Internal Medicine I, University Hospital Frankfurt, Frankfurt, Germany

**Keywords:** fibrosis, cirrhosis, inflammation, angiogenesis, portal hypertension

## Abstract

**Background and Aims:**

Angiogenesis is critically involved in the development of liver fibrosis, portal hypertension (PHT) and hepatocellular carcinoma (HCC). Regorafenib is a novel second-line therapy for HCC, but might also be beneficial in fibrosis and PHT even in absence of HCC. This study investigated the effects of regorafenib in experimental models without HCC.

**Methods:**

Fibrosis (*in vivo* and *in vitro*), inflammation, liver damage (aminotransferases), angiogenesis (matrigel implantation) and *in vivo* systemic and portal hemodynamics were assessed in different mouse and rat models (bile duct ligation, CCl_4_, partial portal vein ligation) after acute and chronic treatment with regorafenib.

**Results:**

Long-term treatment with regorafenib improved portal hypertension most likely due to blunted angiogenesis, without affecting fibrosis progression or regression. Interestingly, acute administration of regorafenib also ameliorated portal hemodynamics. Although regorafenib treatment led to hepatotoxic side effects in long-term treated fibrotic animals, in partial portal vein ligated rats, no liver toxicity due to regorafenib was observed.

**Discussion:**

Regorafenib might be especially suitable as therapy in patients with PHT and preserved liver function.

## INTRODUCTION

In chronic liver disease, activation of contractile myofibroblasts with excessive extracellular matrix production leads to liver fibrosis and thereby increased hepatic vascular resistance [1, 2]. Structural changes in the liver architecture induce intra- and extrahepatic angiogenesis, which further promotes fibrosis progression and vascular dysfunction [3, 4]. Consequently, a vicious cycle is established that deteriorates portal hypertension, the prerequisite for potentially life threatening events, such as variceal bleeding, ascites and hepatorenal syndrome [5]. Also, in vascular liver diseases, portal hypertension can develop with similar complications [6]. Meanwhile, in portal hypertension due to advanced fibrosis or vascular liver diseases, hepatocellular carcinoma (HCC) can develop as it shares several common mechanisms, such as inflammation and/or angiogenesis [6–8]. Sorafenib is already well established as therapy for advanced HCC, while regorafenib is recommended as second-line therapy after sorafenib failure [9, 10]. However, even in the absence of HCC, experimental models suggested an effect of sorafenib on portal hypertension explained by reduced angiogenesis and vasoconstriction of splanchnic vessels [11–13]. Nevertheless, only a limited effect of sorafenib has been observed in human cirrhosis [14].

From the pathophysiological point of view, multikinase inhibitors target an abundance of signaling pathways. Apart from vascular endothelial growth factor and platelet derived growth factor, sorafenib also modulates Transforming protein RhoA (RHOA) / Rho-associated protein kinase (ROCK) signaling [15, 16]. Regorafenib is a more potent multikinase inhibitor [17], which could be particularly beneficial in patients with portal hypertension and HCC.

This study comprehensively investigated the effects of regorafenib and partly compared them to sorafenib in animals with liver fibrosis and portal hypertension.

## RESULTS

### Role of regorafenib treatment in progression and regression of fibrosis *in vivo*

**Figure 1:**
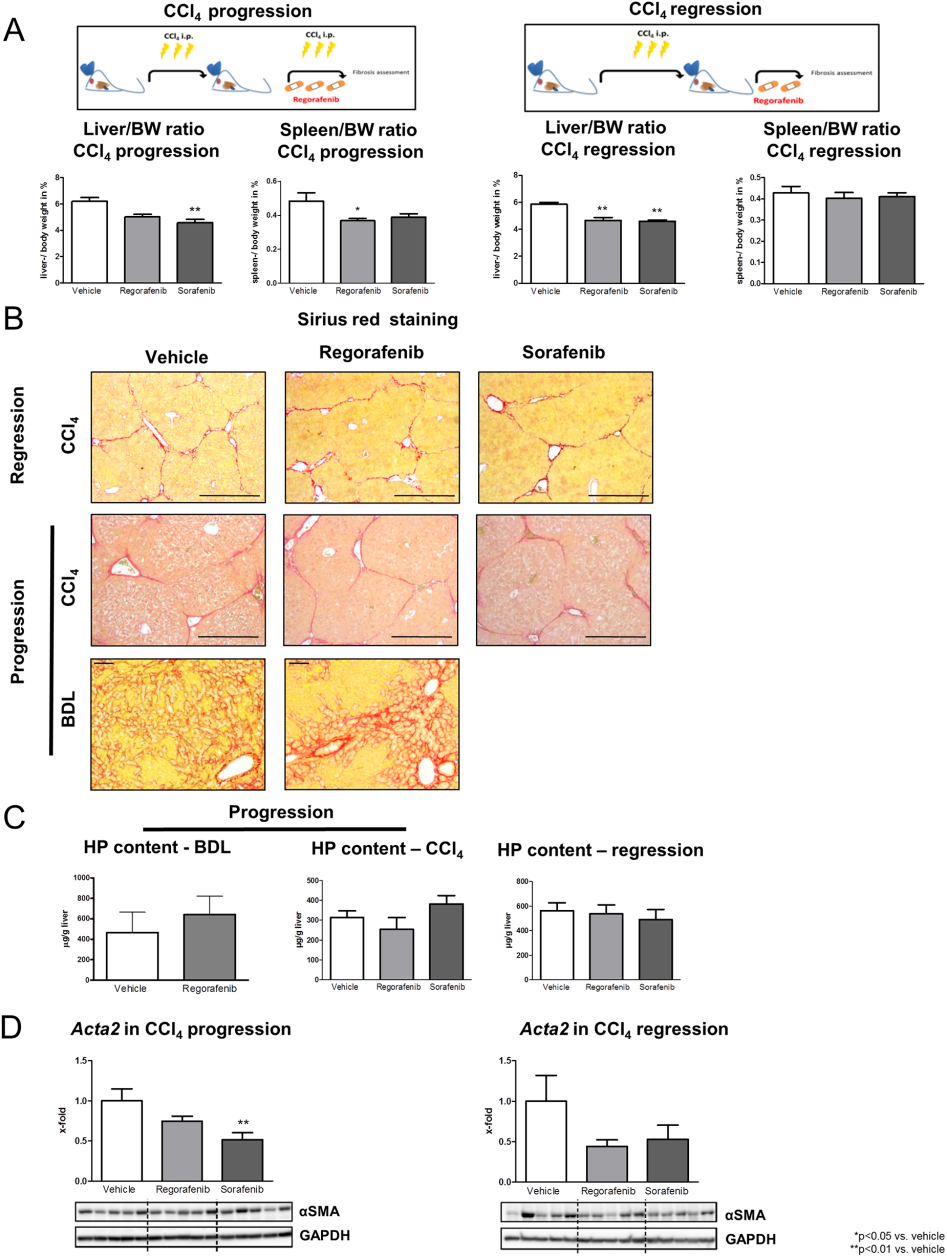
Role of regorafenib treatment in progression and regression of fibrosis *in vivo* **(A)** Liver and spleen bodyweight ratio in the CCl_4_ progression and regression model. (30mg/kg BW sorafenib or regorafenib) ^*^p<0.05 vs. vehicle, ^**^p<0.01 vs. vehicle (Kruskal-Wallis test or Bonferroni post-test). **(B)** Sirius red staining of liver sections as well as **(C)** hepatic hydroxyproline content in BDL rats, in the mouse progression and in the regression model after regorafenib or sorafenib administration (Kruskal-Wallis test or Bonferroni post-test). (BDL: scale bar = 100μm; CCl_4_: scale bar = 400μm) **(D)** *Acta2* mRNA expression and αSMA protein expression after sorafenib and regorafenib treatment in the fibrosis progression and in the regression mouse model ^**^p<0.01 vs. vehicle (Bonferroni post-test). Data are represented as mean +/− SEM.

Liver fibrosis was induced by intraperitoneal (i.p.) injections of carbon tetrachloride (CCl_4_) (twice per week; 17 injections in total) in mice and regorafenib (30mg/kg BW per day), sorafenib (30mg/kg BW per day) or vehicle was administered daily by gavage feeding. Treatment started at the 14^th^ CCl_4_ injection for a further 14 days to determine the effects on fibrosis progression, while for fibrosis regression, treatment started on the last day of CCl_4_ application for six additional days (Figure 1A).

While regorafenib reduced the spleen-bodyweight ratio, sorafenib reduced the liver- bodyweight ratio in the CCl_4_ progression model. By contrast, regorafenib and sorafenib reduced the liver- but not the spleen-bodyweight ratio in the regression mouse model compared to vehicle-treated animals (Figure 1A). Importantly, regorafenib and sorafenib treatment had no direct effect on fibrosis, shown by unchanged liver appearance (Supplementary Figure 1A), Sirius red staining (Figure 1B), hepatic hydroxyproline content (Figure 1C) and expression of profibrotic markers (Figure 1D; Supplementary Figure 1B-1C) in both animal models. Although hepatic mRNA levels of *actin, alpha 2, smooth muscle, aorta* (*Acta2)* were slightly reduced after sorafenib and regorafenib administration, protein levels remained unaltered in both therapeutic approaches (Figure 1C-1D). Furthermore, regorafenib treatment (30mg/kg BW per day; 14 days) had no effect on Actin, aortic smooth muscle (α-SMA) deposition, hydroxyproline content or hepatic profibrotic marker expression in an additional fibrosis progression model (bile duct ligation/BDL in rats) (Figure 1B-1C; Supplementary Figure 1C-1D). Nevertheless, regorafenib treatment significantly reduced hepatic *interleukin 1beta*, *interleukin 6* and *C-X-C motif chemokine ligand 10* mRNA expression in fibrosis regression, while in the progression model, none of these expressions were influenced by regorafenib or sorafenib (Supplementary Figure 1E).

### Effect of regorafenib on HSC activation *in vitro*

**Figure 2:**
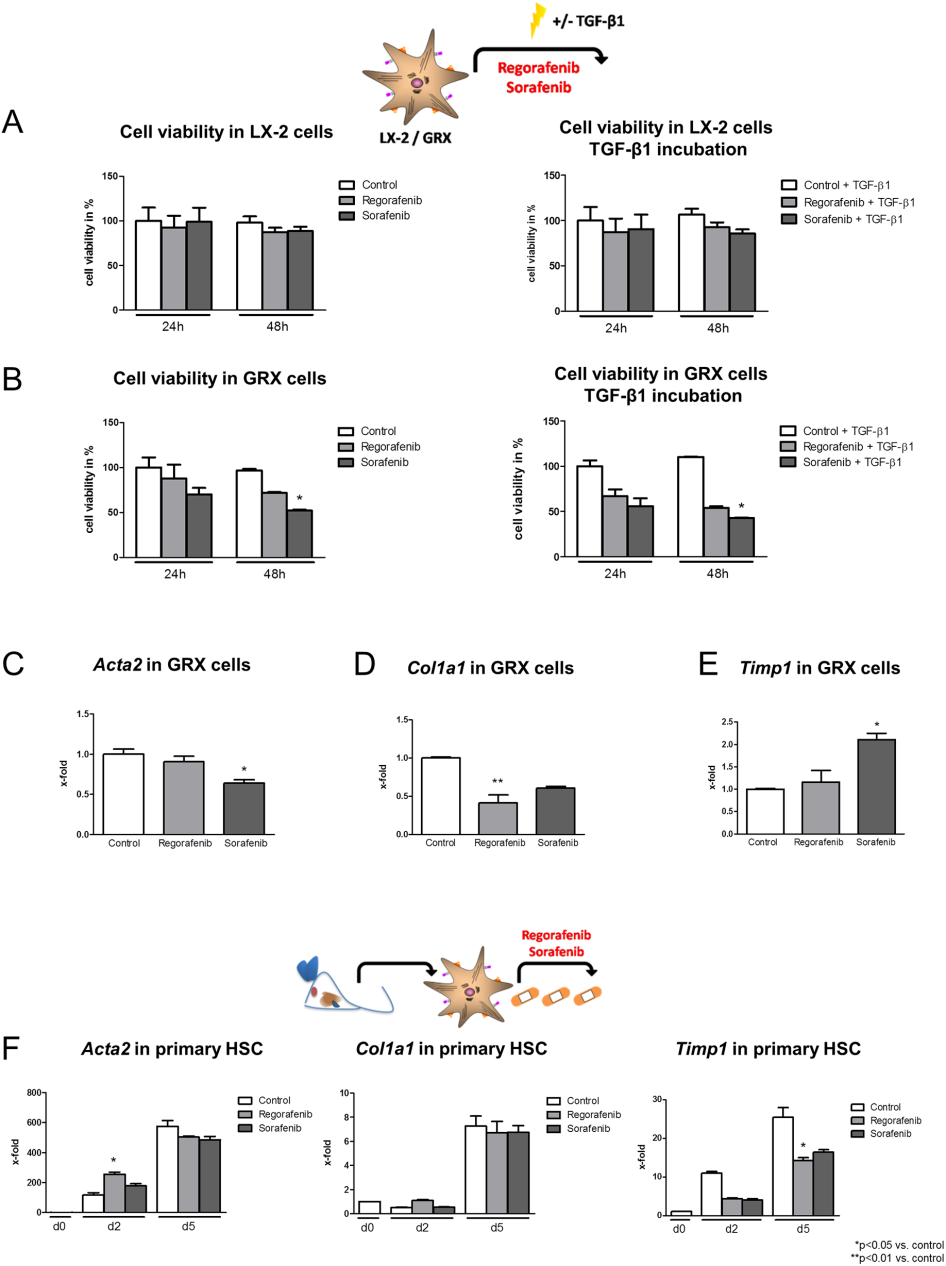
Effect of regorafenib on HSC activation *in vitro* **(A)** Cell viability of human-derived LX-2 cells after 24 h and 48 h incubation with sorafenib or regorafenib (10nmol/ml) alone and after additional incubation with TGF-β1 (4ng/ml). **(B)** Cell viability in murine GRX cells after sorafenib or regorafenib incubation with and without TGF-β1 (20ng/ml) ^*^p=0.05 vs. control (Kruskal-Wallis test). TGF-β1 concentration for murine and human cells was chosen according to previous published literature [44, 45]. mRNA expression of **(C)** *Acta2*, **(D)** *Col1a1* and **(E)** *Timp1* after regorafenib or sorafenib treatment (both 10nmol/ml) in GRX cells and **(F)** primary isolated murine HSC. ^*^p<0.05 vs. control, ^**^p<0.01 vs. control (Kruskal-Wallis test). Data are represented as mean +/− SEM.

Immortalized human (LX-2 cells) and murine (GRX cells) hepatic stellate cells (HSC) were used to analyze the effects of regorafenib and sorafenib (10nmol/ml) *in vitro*. Cell viability was not altered after regorafenib or sorafenib incubation in LX2 cells with and without parallel TGF-β1 (4 ng/ml) stimulation (Figure 2A), while GRX cells displayed a reduced cell viability after 48 h of sorafenib administration with and without parallel TGF-β1 (20ng/ml) activation (Figure 2B).

The mRNA expression of *Acta2, collagen, type I, alpha 1 (Col1a1)* and *tissue inhibitor of metalloproteinase 1* (*Timp1)* was measured in GRX cells 24 h after regorafenib or sorafenib treatment. However, HSC marker expression was only slightly and inconsistently altered after either regorafenib or sorafenib incubation (Figure 2C-2E) and after parallel TGF-β1 stimulation (Supplementary Figure 2A-2C).

Next, the effect of regorafenib and sorafenib on isolated primary murine HSCs was analyzed. HSCs were treated *in vitro* with regorafenib or sorafenib for 12 h before being harvested on day 2 or day 5 and expression of *Acta2*, *Col1a1* and *Timp1* was monitored (Figure 2F). Since (i) regorafenib led to an elevated *Acta2* expression on day 2 but not on day 5, (ii) *Col1a1* remained unaltered by regorafenib and sorafenib compared to the respective controls and (iii) only *Timp1* expression was mildly reduced after regorafenib treatment on day 5, no consistent effect of regorafenib or sorafenib on HSC activation could be observed in this experimental setup (Figure 2F).

In summary, regorafenib had no direct observable effect on HSC activation, fibrosis progression or regression. However, since recent studies showed antiangiogenic properties of multikinase inhibitors, which might be beneficial in chronic liver disease with portal hypertension [12–14], we further analyzed the angiogenic and hemodynamic changes in response to regorafenib.

### Changes in hemodynamics and splanchnic angiogenesis after chronic regorafenib treatment

**Figure 3:**
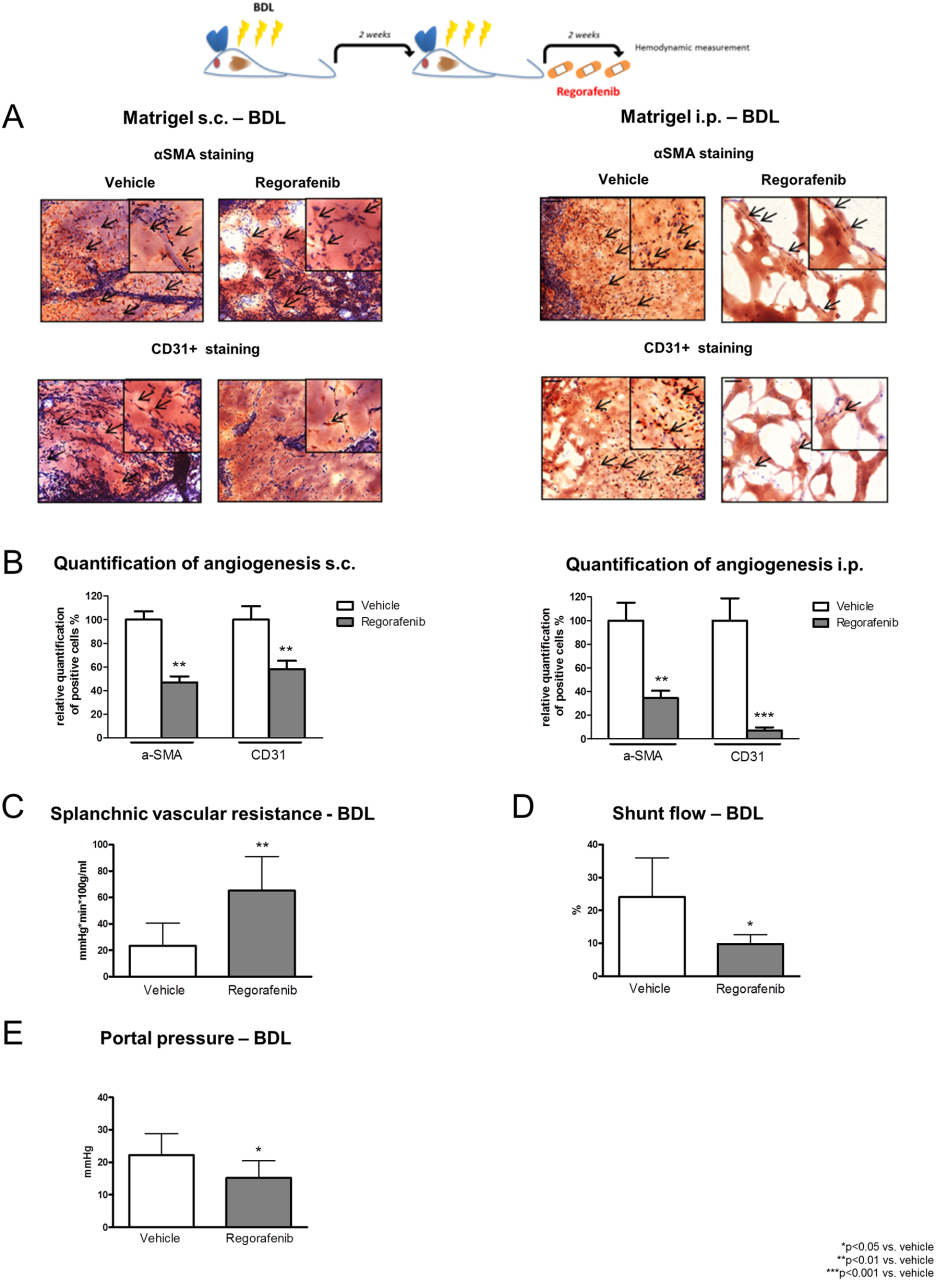
Changes in hemodynamics and splanchnic angiogenesis after two weeks of regorafenib treatment **(A)** αSMA and CD31+ staining of s.c. and i.p. implanted matrigel in BDL rats after vehicle or regorafenib treatment (30mg/kg BW). **(B)** Quantification of angiogenesis in BDL rats treated with regorafenib or vehicle. ^**^p<0.01 vs. vehicle, ^***^p<0.001 vs. vehicle (Mann-Whitney U-test). **(C)** Splanchnic vascular resistance, **(D)** portosystemic collateral blood flow and **(E)** portal pressure after long-term treatment with regorafenib compared to vehicle. ^*^p<0.05 vs. vehicle, ^**^p<0.01 vs. vehicle (Mann-Whitney U-test). Data are represented as mean +/− SEM.

After two weeks of BDL, rats were treated with regorafenib (30mg/kg BW per day) for an additional two weeks by gavage feeding. Then, angiogenesis was assessed *in vivo* using matrigel implantation (subcutaneous: s.c. and intraperitoneal: i.p). At the end of the experiments, matrigel was stained for cluster of differentiation 31+ (CD31+) and α-SMA as marker for endothelial cells (EC) and pericytes. Interestingly, the amount of CD31+ cells (EC) and the amount of α-SMA-positive cells (pericytes) in the s.c. and i.p. implanted matrigel were significantly lower after treatment with regorafenib (Figure 3A-3B).

Since angiogenesis due to new vessel formation reduces vascular resistance, further worsens splanchnic hyperperfusion and aggravates portal hypertension, we measured invasive hemodynamics after completion of the experiments.

In the BDL model, regorafenib treatment significantly increased splanchnic vascular resistance (SpVR) and reduced portosystemic collateral blood flow (SF) compared to vehicle-treated animals (Figure 3C-3D). Furthermore, daily administration of regorafenib significantly reduced portal pressure (PP) compared to vehicle-treated animals without influencing mean arterial pressure (MAP) (Figure 3E, Table 1).

**Table 1:** Hemodynamic changes after regorafenib treatment in BDL rats

	acute regorafenib administration in BDL rats															two weeks of regorafenib administration in BDL rats				
	vehicle (n=5)					10mg/kg regorafenib (n=7)					30mg/kg regorafenib (n=6)					vehicle (n=5)		30mg/kg regorafenib (n=6)		
	before		after			before		after			before		after							
	mean	SEM	mean	SEM	p-value	mean	SEM	mean	SEM	p-value	mean	SEM	mean	SEM	p-value	mean	SEM	mean	SEM	p-value
hepatic vascular resistance (mmHg^*^min^*^100g/ml)	7.159	2.083	7.538	2.017	1.0000	10.15	3.617	7.778	3.314	0.0469^*^	9.73	3.896	7.251	3.359	0.3125	11.80	1.557	4.384	0.8603	0.0019^**^
systemic vascular resistance (mmHg/ml/min/100g)	4.461	1.383	6.058	1.797	0.1250	4.966	0.704	11.61	4.389	0.0273^*^	3.741	0.9523	8.377	1.915	0.0156^*^	7.291	1.438	5.078	0.9296	0.2142
cardiac output (ml/min)	21.06	4.822	13.51	2.11	0.3750	26.35	7.646	17.04	5.579	0.0469^*^	20.06	3.446	17.86	4.057	1.0000	28.05	6.602	25.55	6.806	0.9333
mean arterial pressure (mmHg)																86.00	9.680	94.50	8.122	0.5212

Interestingly, regorafenib treatment had also intrahepatic effects. Hepatic portal vascular resistance (HPVR) was significantly reduced after treatment with regorafenib compared to vehicle. Thus, systemic vascular resistance (SVR) and cardiac output (CO) were not influenced by regorafenib treatment (Table 1).

Taken together, regorafenib reduced splanchnic angiogenesis in liver fibrosis and thereby improved portal hypertension in long-term treated fibrotic animals. This might be the rationale to prevent portal hypertension-related complications in humans. Since the question remained whether a single dose of regorafenib might be beneficial, acute hemodynamic changes were further analyzed in BDL rats.

### Hemodynamic changes after acute regorafenib treatment in BDL rats

**Figure 4:**
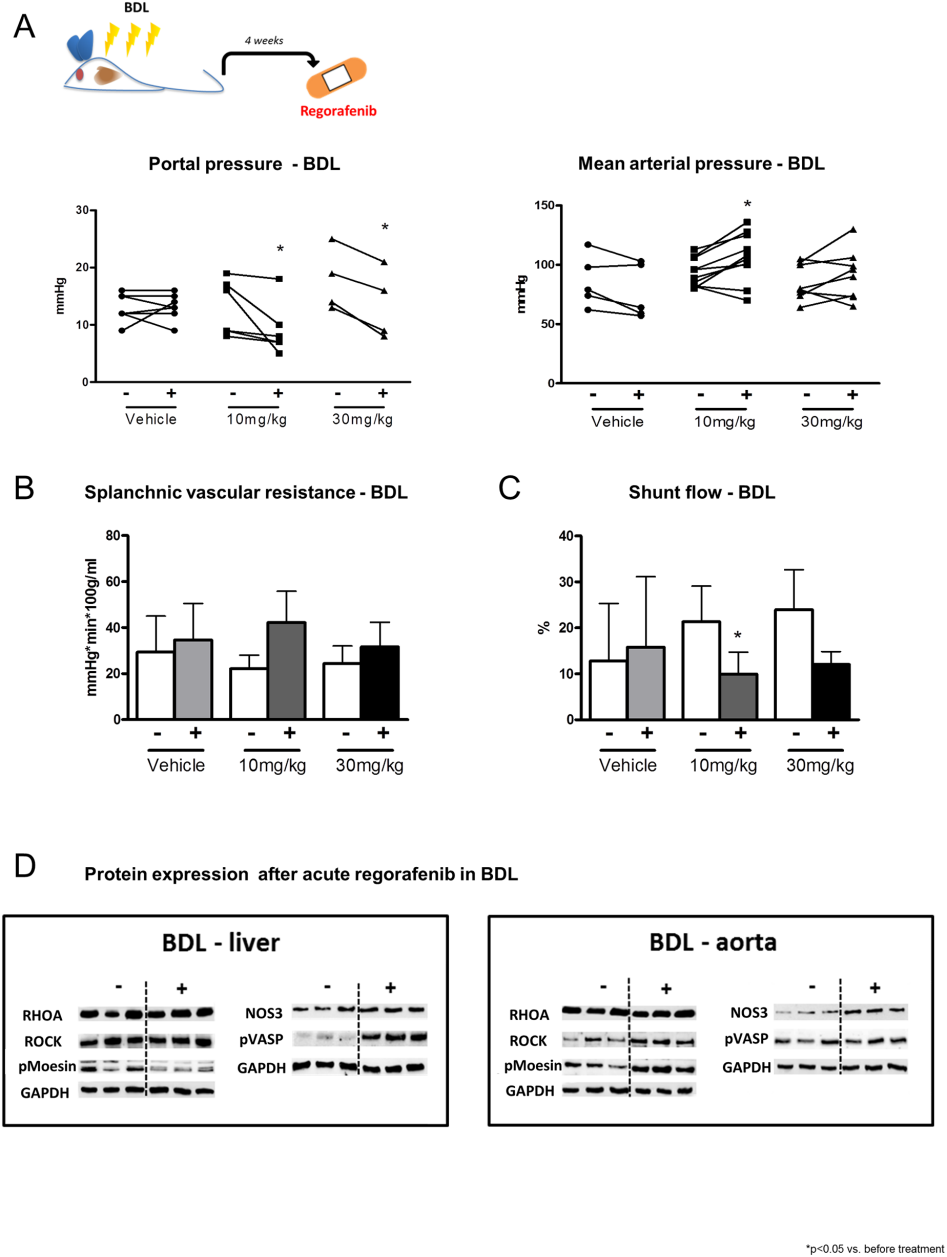
Hemodynamic changes after acute regorafenib treatment **(A)** Portal pressure, mean arterial pressure, **(B)** splanchnic vascular resistance and **(C)** portosystemic collateral blood flow before and after acute administration of vehicle or regorafenib (10mg/kg and 30mg/kg BW) in BDL rats. ^*^p<0.05 vs. before treatment (Mann-Whitney U-test). **(D)** Protein expression of RhoA, ROCK, pMoesin, nitric oxide synthase 3 (NOS3) and pVASP in liver and aortic tissue of BDL rats after regorafenib treatment. GAPDH served as endogenous control. Data are represented as mean +/− SEM.

After induction of liver cirrhosis by BDL for four weeks, a single dose of regorafenib (10mg/kg or 30mg/kg BW) was administered intravenously and hemodynamic changes were assessed. PP was significantly reduced after regorafenib treatment with 10mg/kg and 30mg/kg regorafenib, while MAP was increased (Figure 4A). Both doses of regorafenib slighlty increased SpVR compared to vehicle treatment and 10mg/kg regorafenib significantly reduced SF (Figure 4B-4C). Interestingly, 10mg/kg BW regorafenib treatment significantly reduced HPVR and CO, while SVR was significantly increased compared to vehicle treatment (Table 1). The dose of 30mg/kg BW also reduced HPVR, while CO and SVR were not altered by regorafenib.

These changes were due to reduced ROCK activation assessed by phosphorylated moesin protein and nitric oxide (NO) availability, shown by increased vasodilator-stimulated phosphoprotein (VASP) protein phosphorylation in cirrhotic liver tissue (Figure 4D). Moreover, regorafenib treatment induced ROCK activation, shown by increased moesin protein phosphorylation in aortas of BDL rats, while phosphorylation of VASP remained unchanged (Figure 4D).

In conclusion, acute regorafenib administration also had beneficial effects on portal hemodynamics, which could provide a useful short-term approach in humans with liver fibrosis and portal hypertension.

### Hepatotoxicity of regorafenib and sorafenib

As several reports have highlighted the hepatotoxicity of multikinase inhibitors [18–21] serum liver function markers were determined to further evaluate liver toxicity due to regorafenib treatment.

**Figure 5:**
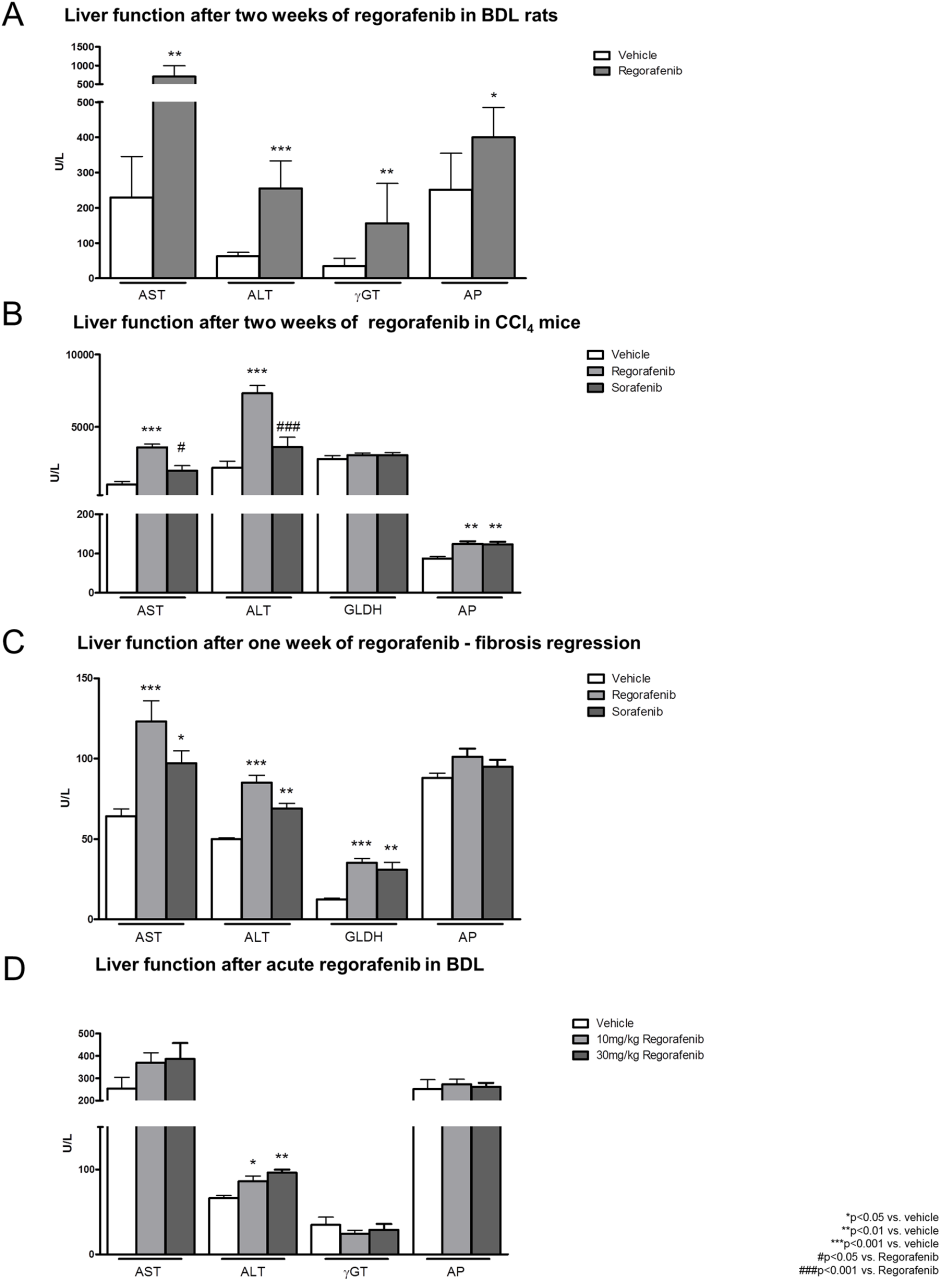
Hepatotoxicity of regorafenib and sorafenib **(A)** Serum liver function markers (AST, ALT, μGT and AP) after long-term treatment with regorafenib (30mg/kg BW) in BDL rats compared to vehicle. ^*^p<0.05 vs. vehicle, ^**^p<0.01 vs. vehicle, ^***^p<0.001 vs. vehicle (Mann-Whitney U-test). **(B-C)** Serum levels of AST, ALT, GLDH and AP after treatment with sorafenib or regorafenib (30mg/kg BW) in fibrosis progression and regression. ^*^p<0.05 vs. vehicle, ^**^p<0.01 vs. vehicle, ^***^p<0.001 vs. vehicle, #p<0.05 vs. regorafenib, ###p<0.001 vs. regorafenib (Kruskal-Wallis test or Bonferroni post-test) **(D)** Levels of AST, ALT, γGT and AP after acute administration of regorafenib (10mg/kg, 30mg/kg BW) in BDL rats compared to vehicle. ^*^p<0.05 vs. vehicle, ^**^p<0.01 vs. vehicle (Mann-Whitney U-test). Data are represented as mean +/− SEM.

After two weeks of treatment, serum levels of alanine transaminase (ALT), aspartate transaminase (AST), γ-glutamyltransferase (γ-GT) and alkaline phosphatase (AP) were considerably increased in regorafenib-treated BDL rats (30mg/kg BW) compared to vehicle (Figure 5A). Comparable results were observed in the mouse fibrosis progression model (30mg/kg BW regorafenib) and less pronounced in the fibrosis regression model (30mg/kg BW regorafenib). Thus, AST, ALT and AP were significantly increased in the fibrosis progression model after regorafenib treatment compared to vehicle controls, while AST, ALT and glutamate dehydrogenase (GLDH) serum levels were increased in the fibrosis regression model (Figure 5B-5C). Furthermore, sorafenib slightly increased serum AP levels compared to vehicle in the fibrosis progression model and AST, ALT and GLDH in the fibrosis regression model (Figure 5B-5C).

Repeated regorafenib administration resulted in hepatotoxic effects, shown by increased levels of serum function markers. However, as it is unclear whether this is an acute phenomenon or whether it depends on the cumulative dose, we measured serum function markers after acute regorafenib administration in BDL rats (4 weeks).

Interestingly, serum levels of AST, γ-GT and AP remained unchanged after acute single administration of increasing doses (10mg/kg; 30mg/kg) of regorafenib compared to vehicle in BDL rats, while ALT levels were just slightly increased (Figure 5D).

These data further support the hypothesis that regorafenib is useful for therapeutic acute short-term approaches. Nevertheless, regorafenib is mainly metabolized in the liver and thereby predestinates for hepatotoxic side effects. While HCC is frequently associated with portal vein thrombosis, this rarely occurs in the absence of cirrhosis. Furthermore, regorafenib is also used in colon and intestinal stromal cancer, which is possibly associated with portal vein thrombosis. For this group of patients, an anti-angiogenic, antihypertensive drug could be very beneficial. Therefore, we tested the hypothesis whether regorafenib also improves portal hypertension and angiogenesis in a model of subtotal portal vein ligation mimicking partial portal vein thrombosis.

### Effects of regorafenib in an animal model of portal vein obstruction

**Figure 6:**
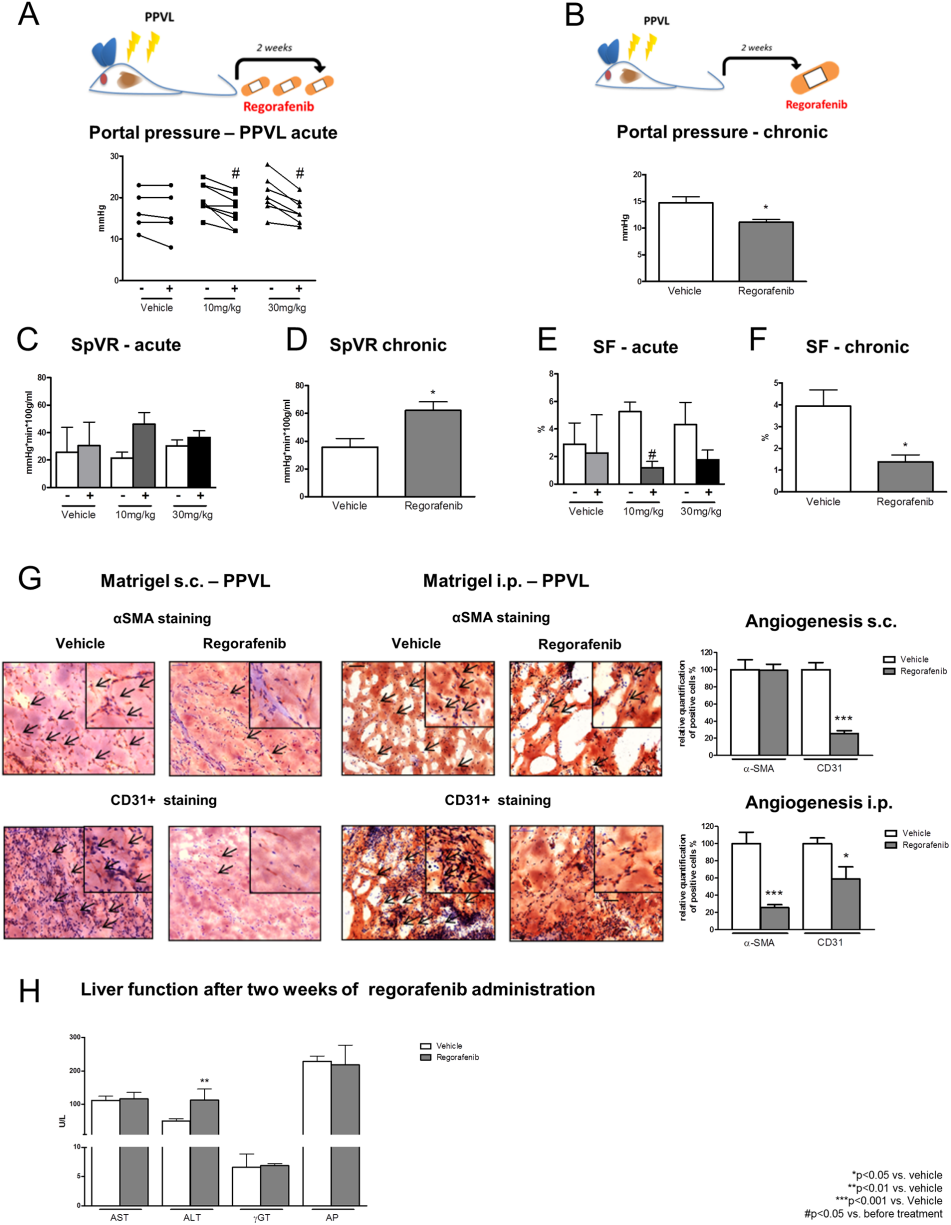
Effects of regorafenib in an animal model of portal vein obstruction **(A-B)** Portal pressure in PPVL rats after acute (10mg/kg and 30mg/kg BW) and long-term treatment (30mg/kg) with regorafenib or vehicle. ^*^p<0.05 vs vehicle, #p<vs. before treatment (Mann-Whitney U-test). **(C-D)** Splanchnic vascular resistance and **(E-F)** portosystemic collateral blood flow in acute and long-term treated PPVL rats compared to vehicle treatment. ^*^p<0.05 vs vehicle, #p<0.05 vs. before treatment (Mann-Whitney U-test). **(G)** αSMA and CD31+positive cells in s.c. and i.p. implanted matrigel of PPVL rats treated with regorafenib. ^*^p<0.05 vs. vehicle, ^***^p<0.001 vs. vehicle (Mann-Whitney U-test). **(H)** ALT, AST, μGT and AP serum levels after long-term treatment with regorafenib in PPVL rats. ^**^p<0.01 vs. vehicle (Mann-Whitney U-test). Data are represented as mean +/− SEM.

Partial portal vein ligation (PPVL) was performed for two weeks in wild type rats. Regorafenib was administered as a single dose (10mg/kg BW; 30mg/kg BW) and acute hemodynamic changes were measured. In another set of PPVL rats, regorafenib was administered daily for two weeks (30mg/kg per day) by gavage feeding. Acute and chronic regorafenib treatment significantly reduced PP in all treated animals, while it remained unchanged after vehicle treatment (Figure 6A-6B). MAP was not influenced by acute and chronic regorafenib administration (Table 2).

**Table 2:** Hemodynamic changes after regorafenib administration in PPVL rats

	acute regorafenib administration in PPVL rats															two weeks of regorafenib administration in PPVL rats				
	vehicle (n=5)					10mg/kg regorafenib (n=6)					30mg/kg regorafenib (n=4)					vehicle (n=7)		30mg/kg regorafenib (n=5)		
	before		after			before		after			before		after							
	mean	SEM	mean	SEM	p-value	mean	SEM	mean	SEM	p-value	mean	SEM	mean	SEM	p-value	mean	SEM	mean	SEM	p-value
cardiac output (ml/min)	29.92	9.493	12.13	0.7631	0.1563	42.70	14.00	24.22	6.047	0.0938	38.16	18.52	22.92	7.422	0.2500	32.83	6.078	40.69	9.637	0.4908
hepatic vascular resistance (mmHg^*^min^*^100g/ml)	5.046	1.531	4.775	1.281	1.0000	6.336	1.511	4.901	0.9297	0.6875	6.696	2.498	6.693	2.925	1.0000	7.036	0.7529	5.481	0.7044	0.1622
systemic vascular resistance (mmHg/ml/min/100g)	4.549	1.271	7.930	0.9028	0.1833	3.302	1.469	7.323	2.052	0.0313^*^	3.272	1.195	3.690	1.295	0.6250	5.597	1.537	4.751	0.8393	0.6594
mean arterial pressure (mmHg)	97.17	7.097	103.00	5.672	0.0975	89.00	6.294	88.86	4.284	1.0000	108.80	9.196	98.50	7.194	0.1859	117.30	5.027	112.00	5.134	0.4765

SpVR was slightly increased after regorafenib treatment, whereas SF was significantly reduced in PPVL rats treated with 10mg/kg BW regorafenib (Figure 6C, 6E). Furthermore, SVR was significantly increased after administration of 10mg/kg regorafenib, while 30mg/kg had no significant influence on SVR (Table 2).

After two weeks of regorafenib administration, SF was significantly reduced compared to vehicle treatment and SpVR was significantly increased in the regorafenib-treated animals (Figure 6D, 6F). HPVR and CO were not altered by acute regorafenib treatment (Table 2) and no effects were observed in HPVR, SVR and CO in animals treated for two weeks (Table 2).

In summary, acute regorafenib administration not only improved cirrhotic portal hypertension, but also hemodynamic circulation in an animal model mimicking portal vein thrombosis.

Next, angiogenesis was assessed by matrigel staining for α-SMA and CD31. Similar to the data obtained from BDL matrigel, the total amount of infiltrating EC and pericytes was significantly reduced in s.c. and i.p. implanted matrigel after treatment with regorafenib compared to the vehicle-treated PPVL rats (6G). Interestingly, except for increased ALT levels, liver serum markers remained unchanged during acute (single doses of 10mg/kg or 30mg/kg BW regorafenib) and chronic regorafenib (30mg/kg BW) administration (Figure 6H).

PPVL rats benefitted from acute as well as chronic regorafenib treatment through an inhibition of angiogenesis without the previously described toxic side effects.

## DISCUSSION

**Figure 7:**
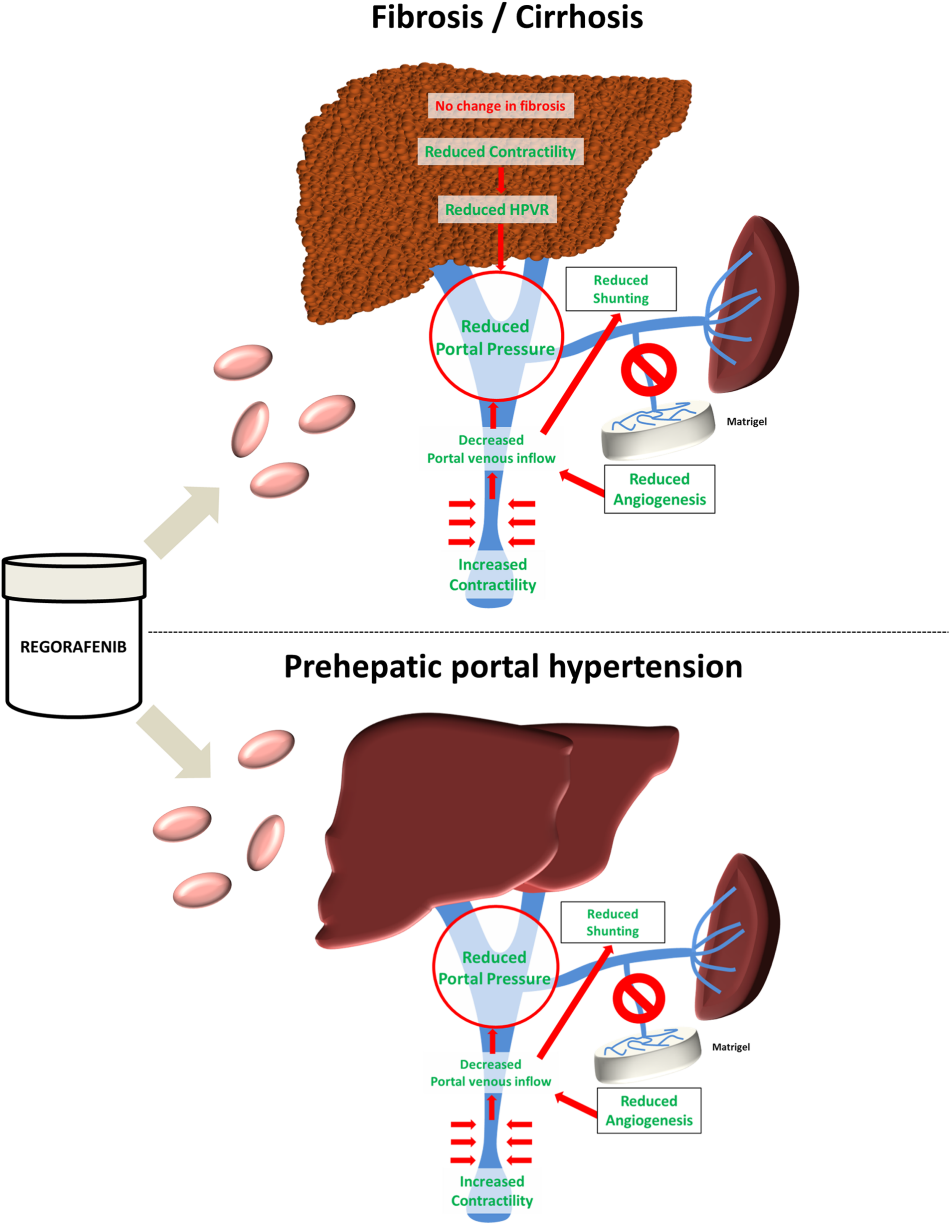
Graphical abstract Treatment with the multikinase inhibitor regorafenib led to reduced contraction and hepatic vascular resistance in experimental liver cirrhosis without a major impact on fibrosis. Still, regorafenib significantly reduced angiogenesis, portosystemic collateral blood flow and shunting and thereby portal pressure. By contrast, in a model of prehepatic portal hypertension, regorafenib improved portal hypertension and reduced angiogenesis without hepatotoxic side-effects.

This study shows that the multikinase inhibitor regorafenib reduces angiogenesis and improves portal hypertension in different experimental models of liver fibrosis and portal vein obstruction (Figure 7).

In recent years, regorafenib was established for the therapy of colorectal cancer and gastrointestinal stromal tumors and was shown to prolong life expectancy in these patients [22–26]. Of note, regorafenib has now been approved as second-line therapy for hepatocellular carcinoma and it is currently the only alternative treatment if patients show disease progression while on sorafenib [9, 10].

Angiogenesis is a common mechanism of these cancers to maintain growth and infiltration. Therefore, the aim of anticancer therapy should be to reduce vessel formation and growth [8]. Moreover, the majority of patients with HCC have concomitant liver fibrosis and portal hypertension. In this scenario, regorafenib might have a dual beneficial effect, considering that angiogenesis is a driver of liver fibrosis [27]. Furthermore, gastrointestinal cancers have thrombotic potential and might lead to portal vein thrombosis, thereby yielding complications for patients and reducing life expectancy [28, 29]. Here, the pleiotropic effects of regorafenib might extend treatment options with better overall management, improving survival and quality of life in these patients.

Considering previous experiences with sorafenib, which was shown to reduce experimental fibrosis and angiogenesis, the investigation of regorafenib in liver disease seems to be a logical consequence [12, 13, 30]. This study shows that regorafenib has strong anti-angiogenic properties, since intrahepatic and extrahepatic angiogenesis was reduced in different animal models of liver fibrosis and portal hypertension. This led to reduced portal venous inflow. Moreover, regorafenib treatment caused splanchnic and systemic vasoconstriction and thereby ultimately reduced portal pressure. Secondary to these effects, the decrease in portal venous inflow also reduced collateral blood flow and shunting. Outstanding hemodynamic changes were also observed after acute treatment with regorafenib due to rebalancing of vasoconstrictive and–dilatory pathways. Thereby, regorafenib improved portal hypertension not only in experimental fibrosis, but also in a model of portal vein obstruction.

However, in our experimental setups, regorafenib and sorafenib displayed no therapeutic effect on the degree of matrix deposition during fibrosis progression or regression. Furthermore, *in vitro* analysis did not show a clear reduction of HSC activation markers after treatment with regorafenib or sorafenib. Several studies have indicated an antifibrotic effect of sorafenib, but inconsistent experimental results can be found in the literature and direct antifibrotic effects, especially in an advanced stage of liver fibrosis, could not be determined with certainty [13, 15, 30–32]. Importantly, sorafenib administration showed antifibrotic properties only in a prophylactic treatment model and in very low doses. In higher doses or later stages of fibrosis, sorafenib had no convincing effect [33]. In our setting, regorafenib showed effects similar to those observed with sorafenib.

Furthermore, hepatotoxicity is frequently noted with regorafenib treatment and cases of liver failure have been reported, with potentially fatal results for the treated patients [18–21]. Our current data are further supporting these findings. Fibrotic animals developed increased levels of serum function markers after chronic treatment with regorafenib (potentially slightly higher than with sorafenib). These effects might be due to the hepatic metabolism of regorafenib by cytochrome P450 3A4. Regorafenib seems to interfere with mitochondrial respiratory chain and thereby induced hepatocyte apoptosis and necroptosis *in vitro*. Taken together, these recently described side-effects might neutralize potential beneficial antifibrotic effects in experimental fibrosis [34, 35]. Importantly, under acute regorafenib treatment, liver function parameters were not markedly influenced, while in the healthy livers of the portal vein ligated animals, regorafenib had no major effect on liver function parameters. This raises the question as to whether regorafenib can be beneficial in the treatment of portal hypertension.

Our data deliver evidence that short-term administration is beneficial in portal hypertension in the presence as well as absence of liver disease without hepatotoxic side effects. Thus, acute treatment might be beneficial when a rapid reduction of portal pressure is needed, while, long-term treatment might be recommended in portal vein thrombosis and preserved liver function, especially if based on preexisting cancer. Nevertheless, there are few major limitations of this study. As recently described, beginning and duration of treatment seem to have a major impact on antifibrotic effects of multikinase inhibitors [33]. This is especially the case for the fibrosis regression model, in which the relatively short observation time during potent heterogenic fibrosis regression might cover antifibrotic effects. Moreover, the used doses of regorafenib might be responsible for the observed hepatotoxic side-effects and might lead to covering of antifibrotic effects. In addition, another major limitation is the lack of human data to confirm our hypotheses in a clinical setting.

However, we see similar effects of regorafenib in different models of liver fibrosis and portal hypertension and in different animal species. In this context, the present study possibly provides important rationales for patient selection, especially since these therapies are very expensive and might culminate in side effects [36].

In conclusion, this study shows that regorafenib could be especially beneficial in patients with preserved liver function and portal hypertension.

## MATERIALS AND METHODS

### Reagents

Regorafenib and sorafenib were kindly provided by Bayer AG, Leverkusen, Germany. Compounds were dissolved in an aqueous solution of 34% of 1,2 propylene glycol (1,2 propandiol, #882281, Sigma-Aldrich, Steinheim, Germany), 34% of polyethylene glycol 400 (#81172, Sigma-Aldrich, Steinheim, Germany) and 12% of Kolliphor® P188 (#15759, Sigma-Aldrich, Steinheim, Germany).

### Animals

We used male wild type (WT) Sprague Dawley rats and male C57BL/6J mice in our experiments. The experiments were performed according to guidelines and regulations approved by LANUV, the responsible committee for animal studies in North Rhine-Westphalia. All animals received water and chow *ad libidum*. Sprague Dawley rats were housed in a controlled environment (standard IVC-cage, 12 h light/dark, temperature 22^°^C −24^°^C), and fed standard rat chow (Ssniff, Soest, Germany). The experiments were performed during light cycle.

### Induction of liver fibrosis in mice

Six to seven week old male C57BL/6J mice were used for both CCl_4_-induced liver fibrosis models. In the liver fibrosis progression model, mice were injected intraperitoneally with 0.6ml carbon tetrachloride per kg body weight (CCl_4_, Sigma Aldrich, Munich, Germany; mixed with sunflower seed oil) twice a week and 17 times in total. Starting from the 14th CCl_4_ injection, mice were treated daily via oral gavage with either regorafenib (30mg/kg body weight) or sorafenib (30mg/kg body weight) for 14 days.

In the liver fibrosis regression model, mice were injected intraperitoneally with CCl_4_ (0.6ml/kg body weight) twice a week and 17 times in total. Regorafenib or sorafenib was administrated via oral gavage for six days, starting on the last day of CCl_4_ injection. Control mice were daily gavaged with vehicle solution (6μl/g body weight) for the respective treatment period.

### Cholestatic model of fibrosis

Bile duct ligation (BDL) was performed in WT rats with an initial body weight (BW) of 180-200g as described previously [37, 38]. Experiments were carried out four weeks after BDL. Rats received either a single intravenous dose of regorafenib (10mg/kg BW or 30mg/kg BW) or daily oral doses of regorafenib (30mg/kg BW per day) for two weeks before sacrifice. Vehicle treatment served as control (1ml/kg BW).

### Partial portal vein ligation (PPVL)

PPVL was performed in WT rats with an initial BW of 180-200g as described previously [39, 40]. Experiments were carried out two weeks after PPVL. Rats received either a single intravenous dose of regorafenib (10mg/kg BW or 30mg/kg BW) after two weeks of PPVL or daily oral doses of regorafenib (30mg/kg BW per day) for two weeks before sacrifice. Vehicle treatment served as control (1ml/kg BW).

### *In vivo* hemodynamic experiments

*In vivo* hemodynamic studies were performed in BDL and PPVL rats as described previously [41]. The regorafenib effect was assessed using invasive continuous measurements of mean arterial pressure (MAP) and portal pressure (PP).

### Microsphere technique

To investigate hemodynamics, the colored microsphere technique was performed as described previously [41], whereby 300,000 systemic (red/yellow) microspheres (15μm diameter, Triton-Technologies, San Diego, USA) were injected in the left ventricle. Mesenteric portal-systemic shunt volume was estimated by injection of 150,000 microspheres (white/blue) in the ileocecal vein [41].

### Hydroxyproline assay

Hepatic hydroxyproline (HP) was measured photometrically in snap-frozen rat (200mg) and mouse (50mg) liver samples as described previously [42–44].

### Cell culture experiments

GRX and LX2 cells were cultured in DMEM in 10% FCS and penicillin/streptomycin as previously described [44, 45]. The murine immortalized HSC-cell line GRX was obtained from Rio de Janeiro Cell Bank (PABCAM, Federal University, Rio de Janeiro, Brazil). For stimulation, cells were seeded for either cell viability in 96-well plates (12,500 cells per well) or RNA expression analysis in 12-well plates (125,000 cells per wells) and were allowed to attach overnight. Stimulation was performed with 10 nmol/ml regorafenib or sorafenib (stock solution dissolved in DMSO; 0.1% DMSO in final solution) in medium that contained 2% FCS for GRX cells and 5% FCS for LX2 cells, according to the literature [44, 46]. Additional TGF stimulation was performed according to the literature with 20ng/ml TGF-β1 (#100-21, PeproTech, Rocky Hill, USA) for GRX cells and 4ng/ml for LX2 cells [44, 45]. Cells incubated in DMEM supplemented with either 5% (LX2) or 2% FCS (GRX) and 0.1% DMSO were used as controls. Cells were used at indicated time points for the respective analysis. Primary HSC were isolated and pooled from three 42-week old C57Bl6 mice via iohexol gradient with subsequent FACS sorting as described previously [47]. For RNA expression analysis, 112,500 cells were seeded out in 12-well plates and cultured in DMEM medium containing 10% FCS and penicillin/streptomycin for up to five days. Cells were stimulated for 12h with 10 nmol/ml regorafenib or 10nmol/ml sorafenib (culture medium containing 0.1% DMSO) before they were harvested two days or five days after seeding.

### Western blotting

Rat liver samples were processed as previously described using sodium dodecyl sulfate - polyacrylamide gel electrophoresis (SDS-PAGE) gels and nitrocellulose membranes [37, 48]. Ponceau-S staining assured equal protein loading. Glyceraldehyde- 3-phosphate dehydrogenase (GAPDH) served as endogenous control. Membranes were incubated with the respective primary antibody (Supplementary Table 1) and corresponding secondary peroxidase-coupled antibody (Santa-Cruz Biotechnology, Santa Cruz, USA). After enhanced chemiluminescence (ECL, Amersham, UK), digital detection was evaluated using Chemi-Smart (PeqLab Biotechnologies, Erlangen, Germany). Mice liver tissues were homogenized in NP-40 lysis buffer using Bead Ruptor 12 (omni international) to obtain protein lysates. Proteins were separated by SDS-PAGE, transferred to *polyvinylidene difluoride* (PVDF) membrane (Merck Millipore) and analyzed by immunoblotting as previously described [49]. Primary antibodies are listed in Supplementary Table 1. As secondary antibody, anti-mouse HRP (GE Healthcare) was used.

### Quantitative RT-PCR

RNA isolation, reverse transcription and detection by RT-PCR in rats were performed as described previously (18,19) and 18S rRNA served as endogenous control. RNA of mice and HSC samples were isolated using *Direct-zol™ RNA MiniPrep* (Zymo Research Europe GmbH, Freiburg, Germany) in combination with RNase-Free DNase Set (Qiagen) or *NucleoSpin RNA Plus* (Macherey-Nagel, Düren, Germany) according to manufacturer's protocols. RNA was reverse transcribed using either *cDNA synthesis Kit H Plus* (Peqlab, VWR International GmbH, Darmstadt, Germany) or *RevertAid RT Reverse Transcription Kit* (#K1691, Thermo Scientific). 2μl of cDNA was added in a total volume of 10μl containing *Fast-SYBR Green* (Applied Biosystems, Foster City, CA, USA) together with specific primers (Supplementary Table 2 and 3) and qPCR was performed on the *QuantStudio 6 flex* PCR system (Applied Biosystems, Foster City, CA, USA). All results are expressed as 2^−ΔΔCT^ and represent the x-fold increase of gene expression compared to the control group.

### Histological staining

As described previously, paraffin sections, fixed with 4% paraformaldehyde) were stained with H/E and Sirius red, respectively, following a standard protocol for paraffin sections [50]. Images were obtained using a Leica DM 1000 microscope and a Leica EC3 camera in combination with Leica Application Suite.

### Immunohistochemical staining for CD31+ and α-SMA

Staining for CD31+ and α-SMA was performed in cryosections from liver tissue (3μm and 7μm thickness, respectively) as described previously [51–53]. Briefly, after several steps, cryosections were incubated with a mouse anti-SMA antibody (clone 1A4; Sigma-Aldrich, Munich, Germany) or with antibody against CD31+ (ab24590, Abcam, Cambridge, UK). Thereafter, a biotinylated rabbit anti-mouse secondary antibody (Dako, Glostrup, Denmark) was used.

### Serum analysis

Levels of serum ALT, AST, GLDH, AP and μGT activity were measured by standard procedures at the Institute of Clinical Chemistry at University Hospital RWTH Aachen and at the Institute for Clinical Chemistry and Clinical Pharmacology at University Hospital Bonn.

### Statistical analysis

Results are expressed as mean ± SEM unless otherwise indicated. Statistical analysis of two groups was performed with Mann-Whitney-*U* test. Only groups with more than three animals were tested statistically. Comparison of three groups was performed by using one-way Anova with Bonferroni post-test or by using Kruskal-Wallis test with Dunn's post-test. Statistical analyses and graphics were performed using GraphPad Prism 5.0 for Macintosh (Graph-Pad, San Diego, USA). p<0.05 was considered statistically significant.

## SUPPLEMENTARY MATERIALS FIGURES AND TABLES



## Abbreviations

Acta2: *actin, alpha 2, smooth muscle, aorta*
AP: alkaline phosphatase
α-SMA: Actin, aortic smooth muscle
AST: aspartate aminotransferase
BDL: bile duct ligation
BW: bodyweight
CCl_4_: carbon tetrachloride
CD31+: cluster of differentiation 31+
γGT: γ-glutamyltransferase
CO: cardiac output
Col1a1: collagen type 1 alpha 1 chain
EC: endothelial cell
NOS3: nitric oxide synthase 3
GAPDH: glyceraldehyde- 3-phosphate dehydrogenase
GLDH: glutamate dehydrogenase
HCC: hepatocellular carcinoma
HPVR: hepatic portal vascular resistance
HSC: hepatic stellate cell
i.p.: intraperitoneal
MAP: mean arterial pressure
mRNA: messenger ribonucleic acid
NO: nitric oxide
PP: portal pressure
PPVL: partial portal vein ligation
RHOA: Transforming protein RhoA
ROCK: Rho-associated protein kinase
s.c.: subcutaneous
SDS-PAGE: sodium dodecyl sulfate - polyacrylamide gel electrophoresis
SF: portosystemic collateral blood flow
SpVR: splanchnic vascular resistance
SVR: systemic vascular resistance
Timp1: tissue inhibitor of metalloproteinase 1
VASP: vasodilator-stimulated phosphoprotein
WT: wild type

## References

[1] Friedman SL (2008). Mechanisms of hepatic fibrogenesis. Gastroenterology.

[2] Reeves HL, Friedman SL (2002). Activation of hepatic stellate cells--a key issue in liver fibrosis. Front Biosci.

[3] Bosch J, Abraldes JG, Fernández M, García-Pagán JC (2010). Hepatic endothelial dysfunction and abnormal angiogenesis: new targets in the treatment of portal hypertension. J Hepatol.

[4] Bosch J, Groszmann RJ, Shah VH (2015). Evolution in the understanding of the pathophysiological basis of portal hypertension: How changes in paradigm are leading to successful new treatments. J Hepatol.

[5] Sanyal AJ, Bosch J, Blei A, Arroyo V (2008). Portal hypertension and its complications. Gastroenterology.

[6] European Association for the Study of the Liver. Electronic address: easloffice@easloffice.eu (2016). EASL Clinical Practice Guidelines: Vascular diseases of the liver. J Hepatol.

[7] Ripoll C, Groszmann RJ, Garcia-Tsao G, Bosch J, Grace N, Burroughs A, Planas R, Escorsell A, Garcia-Pagan JC, Makuch R, Patch D, Matloff DS, Portal Hypertension Collaborative Group (2009). Hepatic venous pressure gradient predicts development of hepatocellular carcinoma independently of severity of cirrhosis. J Hepatol.

[8] Fernández M, Semela D, Bruix J, Colle I, Pinzani M, Bosch J (2009). Angiogenesis in liver disease. J Hepatol.

[9] Bruix J, Tak WY, Gasbarrini A, Santoro A, Colombo M, Lim HY, Mazzaferro V, Wiest R, Reig M, Wagner A, Bolondi L (2013). Regorafenib as second-line therapy for intermediate or advanced hepatocellular carcinoma: multicentre, open-label, phase II safety study. Eur J Cancer.

[10] Bruix J, Qin S, Merle P, Granito A, Huang YH, Bodoky G, Pracht M, Yokosuka O, Rosmorduc O, Breder V, Gerolami R, Masi G, Ross PJ (2017). Regorafenib for patients with hepatocellular carcinoma who progressed on sorafenib treatment (RESORCE): a randomised, double-blind, placebo-controlled, phase 3 trial. Lancet.

[11] Hennenberg M, Trebicka J, Kohistani Z, Stark C, Nischalke HD, Krämer B, Körner C, Klein S, Granzow M, Fischer HP, Heller J, Sauerbruch T (2011). Hepatic and HSC-specific sorafenib effects in rats with established secondary biliary cirrhosis. Lab Invest.

[12] Reiberger T, Angermayr B, Schwabl P, Rohr-Udilova N, Mitterhauser M, Gangl A, Peck-Radosavljevic M (2009). Sorafenib attenuates the portal hypertensive syndrome in partial portal vein ligated rats. J Hepatol.

[13] Mejias M, Garcia-Pras E, Tiani C, Miquel R, Bosch J, Fernandez M (2009). Beneficial effects of sorafenib on splanchnic, intrahepatic, and portocollateral circulations in portal hypertensive and cirrhotic rats. Hepatology.

[14] Pinter M, Sieghart W, Reiberger T, Rohr-Udilova N, Ferlitsch A, Peck-Radosavljevic M (2012). The effects of sorafenib on the portal hypertensive syndrome in patients with liver cirrhosis and hepatocellular carcinoma--a pilot study. Aliment Pharmacol Ther.

[15] Hennenberg M, Trebicka J, Stark C, Kohistani AZ, Heller J, Sauerbruch T (2009). Sorafenib targets dysregulated Rho kinase expression and portal hypertension in rats with secondary biliary cirrhosis. Br J Pharmacol.

[16] Wilhelm S, Carter C, Lynch M, Lowinger T, Dumas J, Smith RA, Schwartz B, Simantov R, Kelley S (2006). Discovery and development of sorafenib: a multikinase inhibitor for treating cancer. Nat Rev Drug Discov.

[17] Wilhelm SM, Dumas J, Adnane L, Lynch M, Carter CA, Schütz G, Thierauch KH, Zopf D (2011). Regorafenib (BAY 73-4506): a new oral multikinase inhibitor of angiogenic, stromal and oncogenic receptor tyrosine kinases with potent preclinical antitumor activity. Int J Cancer.

[18] Béchade D, Desjardin M, Castain C, Bernard PH, Fonck M (2017). Fatal Acute Liver Failure as a Consequence of Regorafenib Treatment in a Metastatic Colon Cancer. Case Rep Oncol.

[19] Mir O, Brodowicz T, Italiano A, Wallet J, Blay JY, Bertucci F, Chevreau C, Piperno-Neumann S, Bompas E, Salas S, Perrin C, Delcambre C, Liegl-Atzwanger B (2016). Safety and efficacy of regorafenib in patients with advanced soft tissue sarcoma (REGOSARC): a randomised, double-blind, placebo-controlled, phase 2 trial. Lancet Oncol.

[20] Uetake H, Sugihara K, Muro K, Sunaya T, Horiuchi-Yamamoto Y, Takikawa H (2018). Clinical Features of Regorafenib-induced Liver Injury in Japanese Patients From Postmarketing Experience. Clin Colorectal Cancer.

[21] Sacré A, Lanthier N, Dano H, Aydin S, Leggenhager D, Weber A, Dekairelle AF, De Cuyper A, Gala JL, Humblet Y, Sempoux C, Van den Eynde M (2016). Regorafenib induced severe toxic hepatitis: characterization and discussion. Liver Int.

[22] Fan LC, Teng HW, Shiau CW, Tai WT, Hung MH, Yang SH, Jiang JK, Chen KF (2016). Regorafenib (Stivarga) pharmacologically targets epithelial-mesenchymal transition in colorectal cancer. Oncotarget.

[23] Takigawa H, Kitadai Y, Shinagawa K, Yuge R, Higashi Y, Tanaka S, Yasui W, Chayama K (2016). Multikinase inhibitor regorafenib inhibits the growth and metastasis of colon cancer with abundant stroma. Cancer Sci.

[24] Stintzing S (2014). Management of colorectal cancer. F1000Prime Rep.

[25] Grothey A, Van Cutsem E, Sobrero A, Siena S, Falcone A, Ychou M, Humblet Y, Bouché O, Mineur L, Barone C, Adenis A, Tabernero J, Yoshino T (2013). Regorafenib monotherapy for previously treated metastatic colorectal cancer (CORRECT): an international, multicentre, randomised, placebo-controlled, phase 3 trial. Lancet.

[26] Li J, Qin S, Xu R, Yau TC, Ma B, Pan H, Xu J, Bai Y, Chi Y, Wang L, Yeh KH, Bi F, Cheng Y (2015). Regorafenib plus best supportive care versus placebo plus best supportive care in Asian patients with previously treated metastatic colorectal cancer (CONCUR): a randomised, double-blind, placebo-controlled, phase 3 trial. Lancet Oncol.

[27] Shah VH, Bruix J (2009). Antiangiogenic therapy: not just for cancer anymore?. Hepatology.

[28] Regnault H, Emambux S, Lecomte T, Doat S, Dhooge M, Besson M, Dubreuil O, Moryoussef F, Silvain C, Bachet JB, Tougeron D (2018). Clinical outcome of portal vein thrombosis in patients with digestive cancers: A large AGEO multicenter study. Dig Liver Dis.

[29] Riva N, Donadini MP, Dentali F, Squizzato A, Ageno W (2012). Clinical approach to splanchnic vein thrombosis: risk factors and treatment. Thromb Res.

[30] Wang Y, Gao J, Zhang D, Zhang J, Ma J, Jiang H (2010). New insights into the antifibrotic effects of sorafenib on hepatic stellate cells and liver fibrosis. J Hepatol.

[31] Liu C, Yang Z, Wang L, Lu Y, Tang B, Miao H, Xu Q, Chen X (2015). Combination of sorafenib and gadolinium chloride (GdCl3) attenuates dimethylnitrosamine(DMN)-induced liver fibrosis in rats. BMC Gastroenterol.

[32] Sung YC, Liu YC, Chao PH, Chang CC, Jin PR, Lin TT, Lin JA, Cheng HT, Wang J, Lai CP, Chen LH, Wu AY, Ho TL (2018). Combined delivery of sorafenib and a MEK inhibitor using CXCR4-targeted nanoparticles reduces hepatic fibrosis and prevents tumor development. Theranostics.

[33] Hong F, Chou H, Fiel MI, Friedman SL (2013). Antifibrotic activity of sorafenib in experimental hepatic fibrosis: refinement of inhibitory targets, dosing, and window of efficacy in vivo. Dig Dis Sci.

[34] Wang YK, Xiao XR, Xu KP, Li F (2018). Metabolic profiling of the anti-tumor drug regorafenib in mice. J Pharm Biomed Anal.

[35] Paech F, Mingard C, Grünig D, Abegg VF, Bouitbir J, Krähenbühl S (2018). Mechanisms of mitochondrial toxicity of the kinase inhibitors ponatinib, regorafenib and sorafenib in human hepatic HepG2 cells. Toxicology.

[36] Parikh ND, Singal AG, Hutton DW (2017). Cost effectiveness of regorafenib as second-line therapy for patients with advanced hepatocellular carcinoma. Cancer.

[37] Trebicka J, Hennenberg M, Laleman W, Shelest N, Biecker E, Schepke M, Nevens F, Sauerbruch T, Heller J (2007). Atorvastatin lowers portal pressure in cirrhotic rats by inhibition of RhoA/Rho-kinase and activation of endothelial nitric oxide synthase. Hepatology.

[38] Trebicka J, Hennenberg M, Schulze Pröbsting A, Laleman W, Klein S, Granzow M, Nevens F, Zaagsma J, Heller J, Sauerbruch T (2009). Role of β3-adrenoceptors for intrahepatic resistance and portal hypertension in liver cirrhosis. Hepatology.

[39] Resch M, Wiest R, Moleda L, Fredersdorf S, Stoelcker B, Schroeder JA, Schölmerich J, Endemann DH (2009). Alterations in mechanical properties of mesenteric resistance arteries in experimental portal hypertension. Am J Physiol Gastrointest Liver Physiol.

[40] Wiest R, Das S, Cadelina G, Garcia-Tsao G, Milstien S, Groszmann RJ (1999). Bacterial translocation in cirrhotic rats stimulates eNOS-derived NO production and impairs mesenteric vascular contractility. J Clin Invest.

[41] Grace JA, Klein S, Herath CB, Granzow M, Schierwagen R, Masing N, Walther T, Sauerbruch T, Burrell LM, Angus PW, Trebicka J (2013). Activation of the MAS receptor by angiotensin-(1-7) in the renin-angiotensin system mediates mesenteric vasodilatation in cirrhosis. Gastroenterology.

[42] Klein S, Hinüber C, Hittatiya K, Schierwagen R, Uschner FE, Strassburg CP, Fischer HP, Spengler U, Trebicka J (2016). Novel Rat Model of Repetitive Portal Venous Embolization Mimicking Human Non-Cirrhotic Idiopathic Portal Hypertension. PLoS One.

[43] Trebicka J, Hennenberg M, Odenthal M, Shir K, Klein S, Granzow M, Vogt A, Dienes HP, Lammert F, Reichen J, Heller J, Sauerbruch T (2010). Atorvastatin attenuates hepatic fibrosis in rats after bile duct ligation via decreased turnover of hepatic stellate cells. J Hepatol.

[44] Roderburg C, Urban GW, Bettermann K, Vucur M, Zimmermann H, Schmidt S, Janssen J, Koppe C, Knolle P, Castoldi M, Tacke F, Trautwein C, Luedde T (2011). Micro-RNA profiling reveals a role for miR-29 in human and murine liver fibrosis. Hepatology.

[45] Li HY, Ju D, Zhang DW, Li H, Kong LM, Guo Y, Li C, Wang XL, Chen ZN, Bian H (2015). Activation of TGF-β1-CD147 positive feedback loop in hepatic stellate cells promotes liver fibrosis. Sci Rep.

[46] Su TH, Shiau CW, Jao P, Liu CH, Liu CJ, Tai WT, Jeng YM, Yang HC, Tseng TC, Huang HP, Cheng HR, Chen PJ, Chen KF (2015). Sorafenib and its derivative SC-1 exhibit antifibrotic effects through signal transducer and activator of transcription 3 inhibition. Proc Natl Acad Sci U S A.

[47] Bartneck M, Warzecha KT, Tag CG, Sauer-Lehnen S, Heymann F, Trautwein C, Weiskirchen R, Tacke F (2015). Isolation and time lapse microscopy of highly pure hepatic stellate cells. Anal Cell Pathol (Amst).

[48] Klein S, Klösel J, Schierwagen R, Körner C, Granzow M, Huss S, Mazar IG, Weber S, van den Ven PF, Pieper-Fürst U, Fürst DO, Nattermann J, Lammert F (2012). Atorvastatin inhibits proliferation and apoptosis, but induces senescence in hepatic myofibroblasts and thereby attenuates hepatic fibrosis in rats. Lab Invest.

[49] Schneider AT, Gautheron J, Feoktistova M, Roderburg C, Loosen SH, Roy S, Benz F, Schemmer P, Büchler MW, Nachbur U, Neumann UP, Tolba R, Luedde M (2017). RIPK1 Suppresses a TRAF2-Dependent Pathway to Liver Cancer. Cancer Cell.

[50] Roderburg C, Benz F, Vargas Cardenas D, Koch A, Janssen J, Vucur M, Gautheron J, Schneider AT, Koppe C, Kreggenwinkel K, Zimmermann HW, Luedde M, Trautwein C (2015). Elevated miR-122 serum levels are an independent marker of liver injury in inflammatory diseases. Liver Int.

[51] Trebicka J, Racz I, Siegmund SV, Cara E, Granzow M, Schierwagen R, Klein S, Wojtalla A, Hennenberg M, Huss S, Fischer HP, Heller J, Zimmer A (2011). Role of cannabinoid receptors in alcoholic hepatic injury: steatosis and fibrogenesis are increased in CB2 receptor-deficient mice and decreased in CB1 receptor knockouts. Liver Int.

[52] Huss S, Schmitz J, Goltz D, Fischer HP, Büttner R, Weiskirchen R (2010). Development and evaluation of an open source Delphi-based software for morphometric quantification of liver fibrosis. Fibrogenesis Tissue Repair.

[53] Trebicka J, Wix C, von Heydebrand M, Hittatiya K, Reiberger T, Klein S, Schierwagen R, Kristiansen G, Peck-Radosavljevic M, Fischer HP, Møller S, Bendtsen F, Krag A (2014). Expression of vasoactive proteins in gastric antral mucosa reflects vascular dysfunction in patients with cirrhosis and portal hypertension. Liver Int.

