# Supplementary material for: The multikinase inhibitor regorafenib decreases angiogenesis and improves portal hypertension
Source: Oncotarget. 2018 Nov 16;9(90):36220–37. doi: 10.18632/oncotarget.26333 (PMC6281422; doi:10.18632/oncotarget.26333)
Supplement: Supplementary file 1 [file oncotarget-09-36220-s001.pdf]

The multikinase inhibitor regorafenib decreases angiogenesis and improves portal hypertension

SUPPLEMENTARY MATERIALS

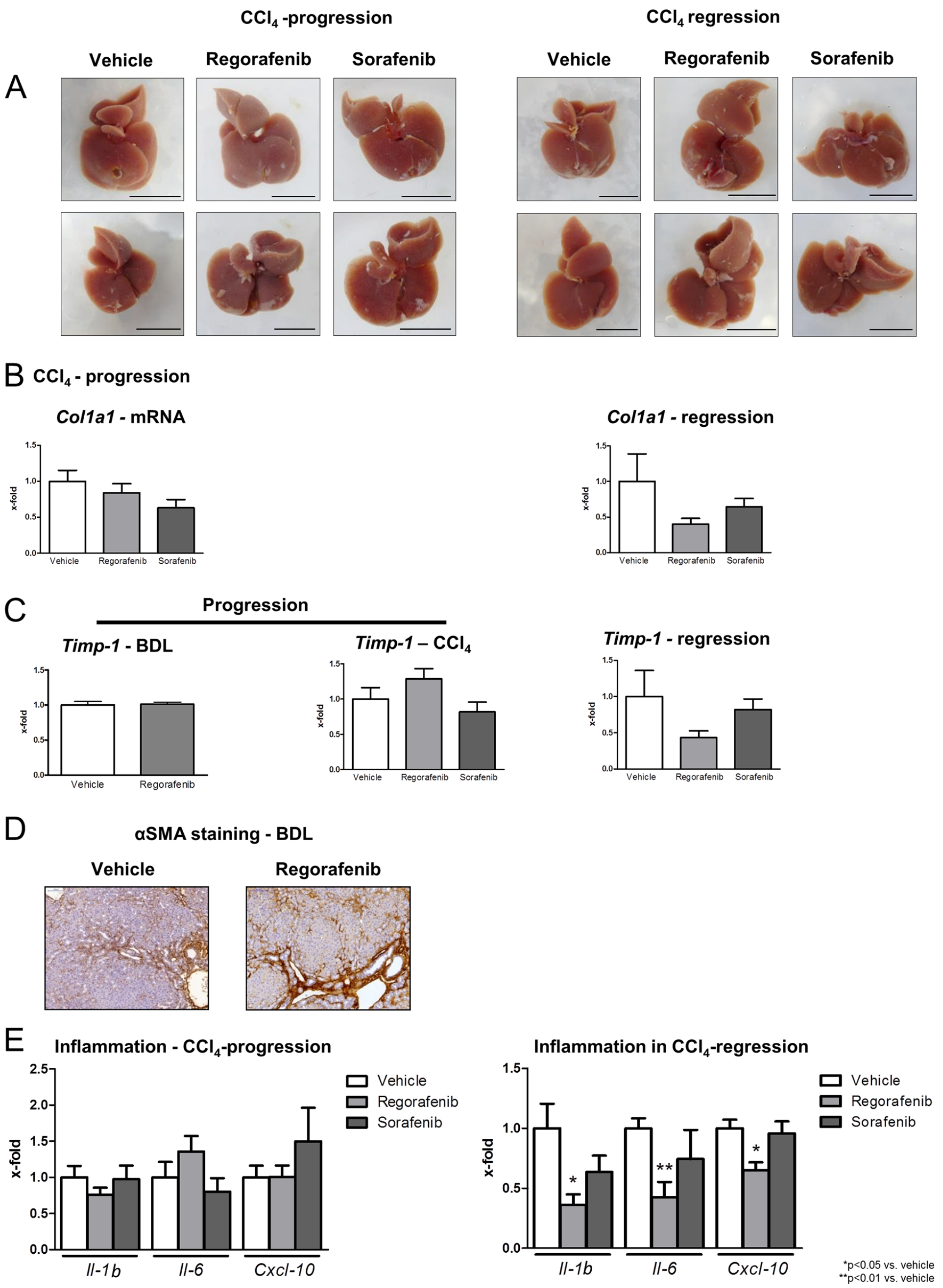

Supplementary Figure 1: (A) Macroscopic images of representative livers shown after treatment with regorafenib or sorafenib as well as **(B)** *Col1a1* relative expression in the CCl_4 progression mice model and the regression mice model. **(C)** Timp1 mRNA expression in BDL rats, in the progression and in the regression mouse model after regorafenib or sorafenib treatment. **(D)** Representative images of αSMA staining in BDL rats treated with vehicle or regorafenib. **(E)** Expression of inflammatory markers *Il1b*, *Il6* and *Cxcl10* after regorafenib treatment in the CCl_4 regression and in the CCl_4 progression model. *p<0.05 vs vehicle, **p<0.01 vs. vehicle (Kruskal-Wallis test or Bonferroni post-test). Data are represented as mean +/- SEM.

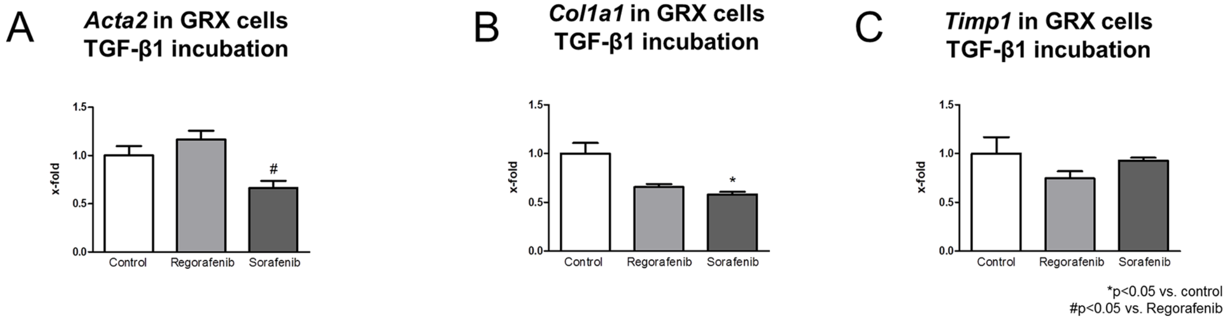

Supplementary Figure 2: **(A)** *Acta2*, **(B)** *Col1a1* and **(C)** *Timp1* mRNA expression (n=4 in each group) in GRX cells co-incubated for 24 h with regorafenib or sorafenib (10nmol/ml) and TGF-β1 (20ng/ml). *p<0.05 vs vehicle, #p<0.05 vs. regorafenib (Kruskal-Wallis test). Data are represented as mean +/- SEM.

Supplementary Table 1: Primary antibodies used for Western blots

Antigen/Origin	Company
αSMA / mouse IgG	Sigma-Aldrich, USA (A2547)
RHOA / mouse IgG	Santa Cruz, USA (sc-418)
ROCK / rabbit IgG	Santa Cruz, USA (sc-5561)
P-Moesin / rabbit IgG	Santa Cruz, USA (sc-12895-R)
NOS3 / mouse IgG	BD, USA (Cat. 610296)
P-VASP / mouse IgG	Merck, USA (#676602)
GAPDH / rabbit IgG	Santa Cruz, USA (sc-25778)
GAPDH / mouse IgG	Bio Rad, USA (MCA 4739)

Supplementary Table 2: Rat-specific primers for qPCR analysis

Gene name	Assay ID	Species
Timp1	Rn00587558_m1	R. norvegicus

Supplementary Table 3: Murine-specific primers for qPCR analysis

**Target**	**forward primer**	**reverse primer**
Acta2	GTC CCA GAC ATC AGG GAG TAA	TCG GAT ACT TCA GCG TCA GGA
Col1a1	GCT CCT CTT AGG GGC CAC T	CCA CGT CTC ACC ATT GGG G
Timp1	GCA ACT CGG ACC TGG TCA TAA	CGG CCC GTG ATG AGA AAC T
Il6	GCTACCAAACTGGATATAATCAGGA	CCAGGTAGCTATGGTACTCCAGAA
Il-1 beta	GCA ACT GTT CCT GAA CTC AAC T	ATC TTT TGG GGT CCG TCA ACT
Cxcl10	CCA AGT GCT GCC GTC ATT TTC	GGC TCG CAG GGA TGA TTT CAA
Gapdh	CCC CAG CAA GGA CAC TGA GCA AGA G	GCC CCT CCT GTT ATT ATG GGG GTC
